# Dermal penetration of 2-phenoxyethanol in humans: in vivo metabolism and toxicokinetics

**DOI:** 10.1007/s00204-024-03938-5

**Published:** 2024-12-24

**Authors:** Elisabeth Eckert, Thomas Jäger, Edgar Leibold, Michael Bader, Thomas Göen, Julia Hiller

**Affiliations:** 1 Institute and Outpatient Clinic of Occupational, Social and Environmental Medicine, Friedrich-Alexander-Universität Erlangen-Nürnberg, Erlangen, Germany; 2 Corporate Health Management, BASF SE, Ludwigshafen, Germany; 3 Bavarian Health and Food Safety Authority, Erlangen, Germany; 4 Product Safety, BASF SE, Ludwigshafen, Germany

**Keywords:** 2-Phenoxyethanol, Skin penetration, Urine, Ethylene glycol monophenyl ether, Biomonitoring, Phenoxyacetic acid

## Abstract

2-Phenoxyethanol (PhE) is an amphiphilic organic compound frequently used as a broad-spectrum preservative in cosmetic products and other consumer goods. PhE is also used as a biocidal component in occupational settings. A previous volunteer study by our working group following oral exposure to PhE showed that PhE is almost completely taken up into the human body followed by an extensive metabolization and fast urinary elimination. However, with respect to the importance of transdermal uptake, we now conducted another volunteer study applying dermal PhE exposure: five volunteers were dermally exposed with 0.4 mg/kg body weight of PhE each on a specified 800 cm^2^ skin area using non-occlusive conditions. Subsequently, blood and urine samples were collected up to 48 h post-exposure. The present study illustrates the fast transdermal uptake of PhE. Following systemic resorption, PhE was extensively metabolized and rapidly eliminated in urine mainly in form of the metabolites PhAA (phenoxyacetic acid) and 4-OH-PhAA (4-hydroxyphenoxyacetic acid) accounting together for over 99% of the renally excreted PhE dose. The absolute urinary recovery rate of PhE was observed to be significantly lower following dermal exposure compared to oral uptake indicating a dermal resorption rate of PhE of about 45% in humans. The present study provides for the first time detailed insights into human biotransformation and toxicokinetics of PhE after dermal exposure, thus establishing a reliable strategy for human biomonitoring of PhE. The here presented results may thus be useful for further toxicokinetic modeling and forward dosimetry.

## Introduction

2-Phenoxyethanol (PhE) is an amphiphilic organic compound, that is used as a broad-spectrum preservative in cosmetic products as well as in other consumer goods, e.g., in disinfectant solutions, vaccine solutions or baby wipes. Its use is limited to a maximum concentration of 1% in cosmetic products in the European Union as stated by Regulation (EC) No. 1223/2009. According to the main areas of PhE application, it may be assumed that the general population is mainly exposed to PhE by the dermal exposure route. Furthermore, PhE has known applications in occupational settings, as it is also used as a biocidal component in metal working fluids for example (ECHA 2024; Hartwig and MAK Commission 2019). Apart from inhalative exposure to PhE at the workplace, dermal penetration of PhE may also play a substantial role in these settings (van Wendel de Joode et al. 2005). In several studies, the significance of dermal PhE absorption was already shown: In in vitro experiments using rat and human skin, mean dermal absorption rates of 64% and 85%, respectively, were observed (reviewed by SCCS 2016). Up to now, however, only one human volunteer study with dermal PhE exposure was described (albeit not sufficiently well): Howes (1988) collected urine samples from four hospitalized patients treated with various amounts of a topical ointment containing 1.2% of PhE (corresponding absolute PhE doses of 240–960 mg). In the urine samples, phenoxyacetic acid (PhAA), as main metabolite of PhE, was solely detected. Recovery in urine showed a high degree of variation and ranged from 8.5% to 42.1% of the applied dose up to 72 h post-exposure (Howes 1988).

As dermal exposure is assumed to be the most relevant route of PhE exposure for humans, SCCS (2016) selected a dermal study in rabbits (most sensitive species to PhE) as a key study for toxicological risk assessment with the critical toxicological effect identified as haematotoxicity, that was observed to be less pronounced in rats and mice. Based on an NOAEL (no observed adverse effect level) of 357 mg/kg body weight (bw) and day, the SCCS derived a health-based guidance value for humans of 14.28 mg/kg bw and day for dermal exposure to PhE.

Due to its widespread use and the potentially high exposure of consumers, PhE was selected as a chemical of interest in a collaboration project for the advancement of human biomonitoring between the German Federal Ministry for the Environment, Nature Conservation, Nuclear Safety and Consumer Protection (BMUV) and the German Chemical Industry Association (VCI) (Kolossa-Gehring et al. 2017). The basic goal of this project is to enable exposure assessment of the general population for substances of potential concern, e.g., due to their toxicity or their relevance for consumer exposure, by establishing reliable human biomonitoring methods and to investigate the biotransformation pathways including the main toxicokinetic parameters of the substances of interest. Thus, novel biomonitoring methods for the determination of PhE and its metabolites in human blood and urine were recently developed by our working group (Jäger et al. 2022, 2024). These procedures enable the determination of PhE in blood and urine as well as the quantification of three PhE metabolites in humans, namely PhAA, 4-hydroxyphenoxyacetic acid (4-OH-PhAA) and 4-hydroxyphenoxyethanol (4-OH-PhE). A former volunteer study conducted by our working group confirmed the suitability of the selected analytes as biomarkers of exposure for PhE: Following oral exposure to PhE with a single dose of 5 mg/kg bw to five volunteers, an average urinary excretion rate of 89.0 ± 11.8% up to 48 h post-exposure was observed, with PhAA and 4-OH-PhAA as the main renally excreted metabolites (Eckert et al. 2024). The almost complete recovery of the applied PhE dose in urine following oral exposure indicates that all relevant human metabolites of PhE have been considered. However, as the main exposure route for humans is assumed to be the dermal route, we now conducted another volunteer study applying dermal exposure to PhE using realistic conditions. In the present study, five volunteers were dermally exposed to PhE (non-occlusively) using a basic ointment containing PhE. The aim of the study was the investigation of PhE metabolism and toxicokinetics in humans following dermal exposure and, in particular, the assessment of the human dermal penetration rate of PhE.

## Materials and methods

### Study population and study design

The study was approved by the local ethics committee of the Friedrich-Alexander-University of Erlangen-Nürnberg, Germany (No. 296_19 B). All participants were informed about the aims and risks of the study and gave their written informed consent to their participation. All subjects were healthy adults who were not occupationally exposed to PhE. The volunteers were instructed to avoid potentially PhE-containing consumer products for at least 3 days prior to PhE administration and during the whole time of sample collection following PhE administration.

The study collective consisted of five volunteers (2 males, 3 females) with a median age of 30 years (range 22–56 years) and a median body weight of 84 kg (range 53–87 kg). All volunteers were exposed dermally to PhE with a single dose of 0.41 ± 0.01 mg PhE/kg bw, corresponding to an absolute dose of 32.2 ± 5.4 mg PhE. The dermal exposure was conducted by non-occlusive application of an ointment (basic formulation) containing 2.0% PhE on the volunteer’s abdomen (with a specified exposure area of 800 cm^2^). The application site was left uncovered for at least 6 h after administration to enable complete absorption of the ointment and to avoid unintended losses, e.g., due to contact with clothing.

The volunteer study took place between August and November 2020. The application scenario was selected to represent an exposure situation as realistic as possible for consumers using PhE-containing cosmetic products. In a worst-case scenario, a full-body application of a cosmetic product (e.g., a body lotion) containing the maximum permitted level of 1% PhE could be assumed. According to the SCCS (2023), the estimated daily applied exposure of body lotion is 7.82 g (123.2 mg/kg bw and day) on a surface area of 15,670 cm^2^ leading to a maximum estimated absolute PhE exposure level of 78.2 mg (1.23 mg/kg bw and day). However, to keep the exposed area as standardized and manageable as possible, while also enabling a reliable determination of minor PhE metabolites, we decided to use a PhE level of 2.0% in the ointment and to expose 800 cm^2^ each of the volunteer’s abdomen. Thus, the selected PhE exposure level still falls safely below the respective DNEL (derived no effect level) which is set at 10.42 mg/kg bw and day for long-term dermal exposure to PhE for the general population (ECHA 2024).

Prior to exposure, each volunteer was asked to deliver one spot-urine sample. Additionally, one blood sample was drawn using EDTA monovettes (pre-exposure samples). The PhE administration was performed early in the morning and the volunteers were instructed to collect all urine samples up to 48 h post-exposure. During the first 8 h after exposure, the volunteers were asked to provide urine samples, if possible, in an hourly interval. All urine samples were weighed to enable an accurate estimation of the individual urine volumes and were stored frozen at −20 °C until analysis. Additionally, blood samples were drawn in regular intervals from each volunteer at 1, 2, 3, 5, 7, 10, 24, 34, and 48 h post-exposure and were also stored frozen at −20 °C until analysis (cf. Table 1).

**Table 1:** Subjects’ characteristics and number of collected samples

Subject no	Age [years], sex	Body weight [kg]	PhE dose [mg (mg/kg bw)]	Sampling time [h]	No. of urine samples	Total urine volume [L]	No. of blood samples
1	56, m	87	35.8 (0.41)	48	30	2.73	14
2	29, f	84	32.8 (0.39)	48	26	3.12	10
3	22, m	87	35.6 (0.41)	48	17	2.65	10
5	30, f	53	22.7 (0.43)	48	19	7.27	10
6	33, f	82	34.3 (0.42)	48	30	3.29	10

M—male, f—female

## Analytical determination of PhE and its metabolites in blood and urine

The analytical determination was carried out according to previously published procedures by Jäger et al. (2022, 2024). Thus, all reagents and chemicals used as well as the applied sample preparation procedures and the instrumentation used are described in detail there.

Briefly, the PhE metabolites PhAA and 4-OH-PhAA were determined using LC–MS/MS analysis following sample preparation using a “dilute-and-shoot” technique and liquid–liquid extraction for urine and blood analysis, respectively. Preliminary experiments indicated that both, PhAA and 4-OH-PhAA, are excreted mainly unconjugated in human urine and that the conjugated forms are present in negligible amounts only. Thus, analyses for these two analytes were done without an additional hydrolysis step (cf. Jäger et al. 2022). Unmetabolized PhE and 4-OH-PhE were determined using GC–PCI-MS/MS analysis following liquid–liquid extraction and silylation. Here, a hydrolysis step was included as it was observed that both PhE and 4-OH-PhE are excreted in significant amounts as conjugates (cf. Jäger et al. 2024). LOQ (limit of quantification) levels of PhAA, 4-OH-PhAA, PhE, and 4-OH-PhE were 10, 20, 1.0, and 0.5 µg/L (urine) and 6, 10, 2.0, and 2.0 µg/L (blood), respectively.

## Data evaluation and statistical analyses

Data evaluation and statistical analyses were done using Microsoft® Excel® 2016 and Origin® 2019, respectively.

Renal excretion rates (R_E_, in μg/h) of each analyte were calculated using the following equation:

$${R}_{E,i}=\frac{{c}_{i}\times {V}_{i}}{{t}_{i}-{t}_{i-1}},$$

where *c*_*i*_ is the analyte concentration in the urine sample (in µg/L), *V*_*i*_ is the volume of the urine sample (in L), *t*_*i*_ is the sampling time of the urine sample after dermal exposure (in h), and *t*_*i-1*_ is the sampling time of the previous urine sample (in h).

Excretion curves were prepared for each study participant and each analyte by plotting the calculated excretion rates against the average time of the sampling period. Mean excretion curves were obtained using default sampling times for each participant and the corresponding mean renal excretion rates at this time point.

The ln-transformed mean excretion curve of each analyte was plotted against the time post-exposure (in h) to obtain the slope (k_el_, elimination rate constant) and the excretion half-life (t_1/2_) in blood and urine, respectively, as follows:

$${t}_{1/2}=\frac{\text{ln}(2)}{\left|{k}_{el}\right|}.$$

Urinary excretion factors (F_UE_) were expressed as PhE dose equivalents (in %) to evaluate the total excretion rate of recovered PhE and its metabolites in urine after 24 h and 48 h, respectively, based on the administered exposure dose of PhE using the following equation:

$${F}_{UE}=\frac{{CE}_{i}}{{M}_{D}}\times 100,$$

where *CE*_*i*_ is the mean cumulative amount of the respective analyte (in µmol) and *M*_*D*_ is the mean administered PhE dose (in µmol), respectively.

## Results

**Fig. 1:**
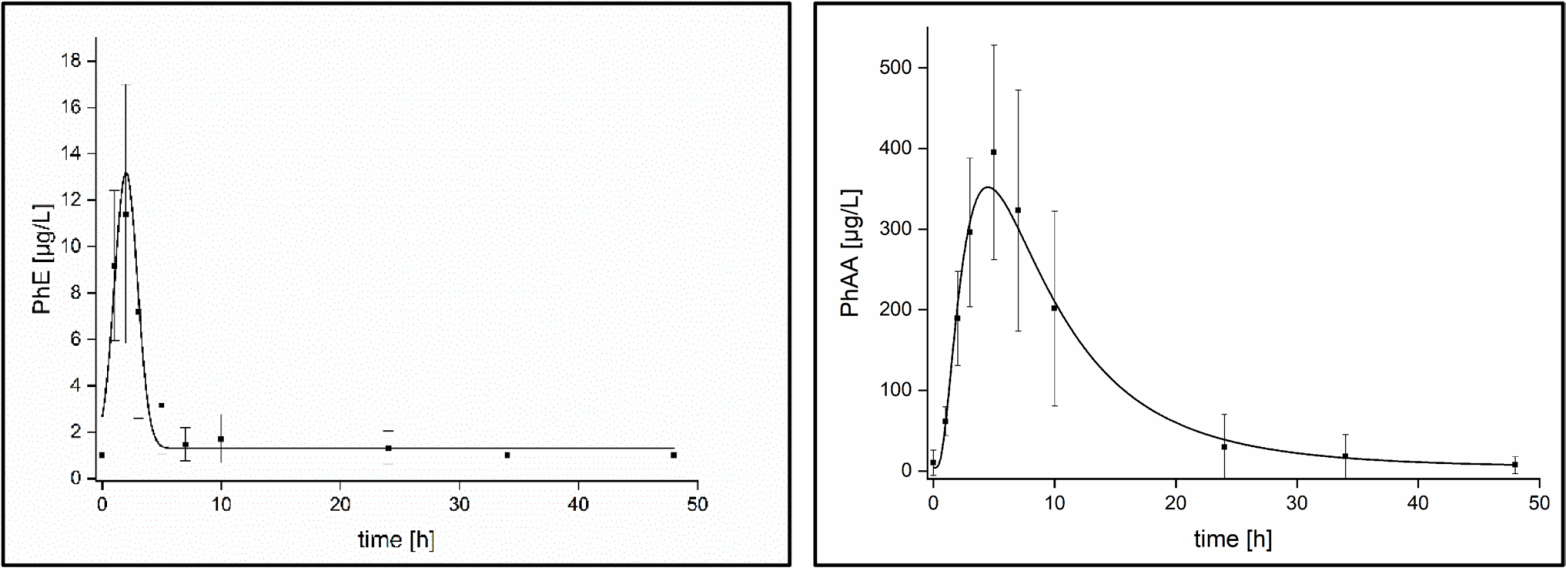
Elimination kinetics of PhE and PhAA in blood following dermal administration of about 0.4 mg/kg body weight PhE to five volunteers

The results for the kinetics of PhE and its metabolites in blood following dermal administration are given in detail in Table 2. Elevated level of unmetabolized PhE was usually observed up to 7 h post-exposure with t_max_ levels between 1 and 2 h post-exposure. Significantly higher blood levels were observed at all time points for the metabolite PhAA with a t_max_ level of 5 h for all volunteers. The metabolite 4-OH-PhAA was found in only one blood sample of volunteer No. 2, while 4-OH-PhE was not found in any of the analyzed blood samples in levels above the LOQ. Figure 1 shows the average elimination kinetics of PhE and its main metabolite PhAA in blood.

**Table 2:** PhE elimination kinetics in blood following dermal PhE administration to five volunteers

Analyte	c_max_ [µg/L]	t_max_ [h]	t_1/2_ [h]
PhE	11.7 ± 5.3	1.6 ± 0.5	1.5 ± 0.7
PhAA	396 ± 133	5.0	3.1 ± 1.9
4-OH-PhAA	109 ^a^	5.0 ^a^	n.a. ^a^

^a^only one volunteer showed elevated levels of 4-OH-PhAA in blood (one sample only)

**Fig. 2:**
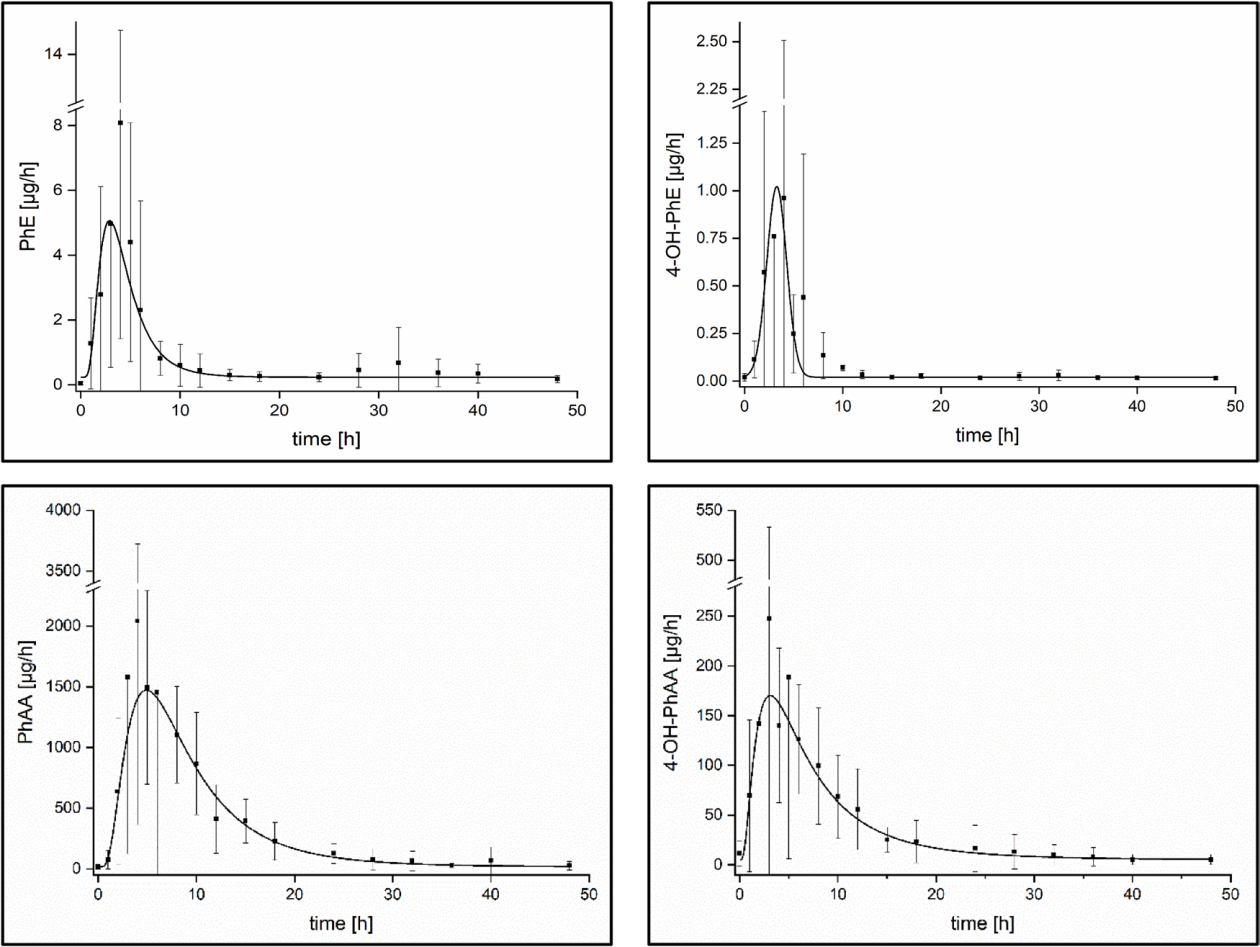
Urinary excretion kinetics of PhE and its metabolites following dermal administration of about 0.4 mg/kg body weight PhE to five volunteers

In the urine samples of all volunteers, unmetabolized PhE as well as all three PhE metabolites were detected following dermal PhE exposure (cf. Table 3). The main metabolites in urine were observed to be PhAA and 4-OH-PhAA for all volunteers with an average relative share of 89.8% and 9.9%, respectively. Unmetabolized PhE and the metabolite 4-OH-PhE were determined in significantly lower levels in the urine samples with relative shares of < 0.5% each. The absolute recovery in urine up to 48 h following dermal exposure amounted to 44.9 ± 10.0% for all analytes with average conversion factors of 40.4% and 4.4% for the main metabolites PhAA and 4-OH-PhAA, respectively. PhAA and 4-OH-PhAA showed delayed t_max_ levels and longer half-lives in direct comparison to PhE itself and to the metabolite 4-OH-PhE (cf. Table 3). For all analytes, distinct excretion kinetics were observed, as shown in Fig. 2.

**Table 3:** Renal excretion kinetics of PhE following dermal administration of about 0.4 mg PhE/kg bw to five volunteers

Analyte	R_E,max_ [µg/h]	t_max_ [h]	t_1/2_ [h]	F_UE_ [24 h, %]	F_UE_ [48 h, %]	rel. analyte share [%]
PhE	8.1 ± 6.6	3.5 ± 0.9	2.5 ± 1.1	0.09 ± 0.07	0.12 ± 0.11	0.3 ± 0.3
4-OH-PhE	0.83 ± 0.82	2.8 ± 0.9	1.6 ± 1.0	0.01 ± 0.01	0.01 ± 0.01	0.02 ± 0.02
PhAA	2477 ± 1,138	5.7 ± 2.6	4.4 ± 1.9	37.1 ± 11.0	40.4 ± 9.8	89.8 ± 4.8
4-OH-PhAA	227 ± 161	7.0 ± 2.3	4.3 ± 0.6	3.9 ± 1.6	4.4 ± 2.2	9.9 ± 4.9
Sum				41.1 ± 11.0	44.9 ± 10.0	100.0

The present study thus illustrates the fast transdermal uptake of PhE into the human body, shown by the short t_max_ of PhE in blood of merely 1.6 h on average. Following systemic resorption, PhE is extensively metabolized and fastly eliminated in the urine. The main metabolite PhAA was observed in blood at a c_max_ level about 30-fold higher compared to unmetabolized PhE. As expected, a temporal delay in the blood elimination kinetics was observed for the metabolite PhAA with a t_max_ level of 5 h and a longer half-life of on average 3.1 h compared to PhE itself. In urine, all four analytes investigated could be determined following dermal exposure to PhE, with unmetabolized PhE and 4-OH-PhE showing average t_max_ levels of 3.5 and 2.8 h, respectively, and rather short half-lives of about 2 h each. Distinctly higher urinary levels, however, were observed for PhAA and hydroxylated PhAA that showed longer half-lives of about 4 h each and delayed average t_max_ levels of 5.7 and 7.0 h, respectively. In sum, a dermal conversion factor of on average 45% was observed up to 48 h after dermal exposure to PhE, with PhAA and 4-OH-PhAA as the main urinary metabolites accounting for a conversion factor of 40.4% and 4.4% on average, respectively.

## Discussion

The present study illustrates similar urinary elimination kinetics of PhE and its metabolite 4-OH-PhE, whereas the metabolites PhAA and 4-OH-PhAA are characterized by longer half-lives and delayed urinary t_max_ levels indicating that the formation of the acid metabolites requires significantly more time than the hydroxylation of the aromatic moiety of PhE. The biotransformation of PhE to phenoxyacetic acid metabolites is a two-step oxidation process catalyzed by cytosolic alcohol dehydrogenase (ADH) and aldehyde dehydrogenase (ALDH), both situated particularly in the liver but are also found in the skin (SCSS 2016). 4-OH-PhE may therefore play a role as an intermediate in the formation of 4-OH-PhAA, but was found itself in consistently low urinary levels in all volunteers.

**Fig. 3:**
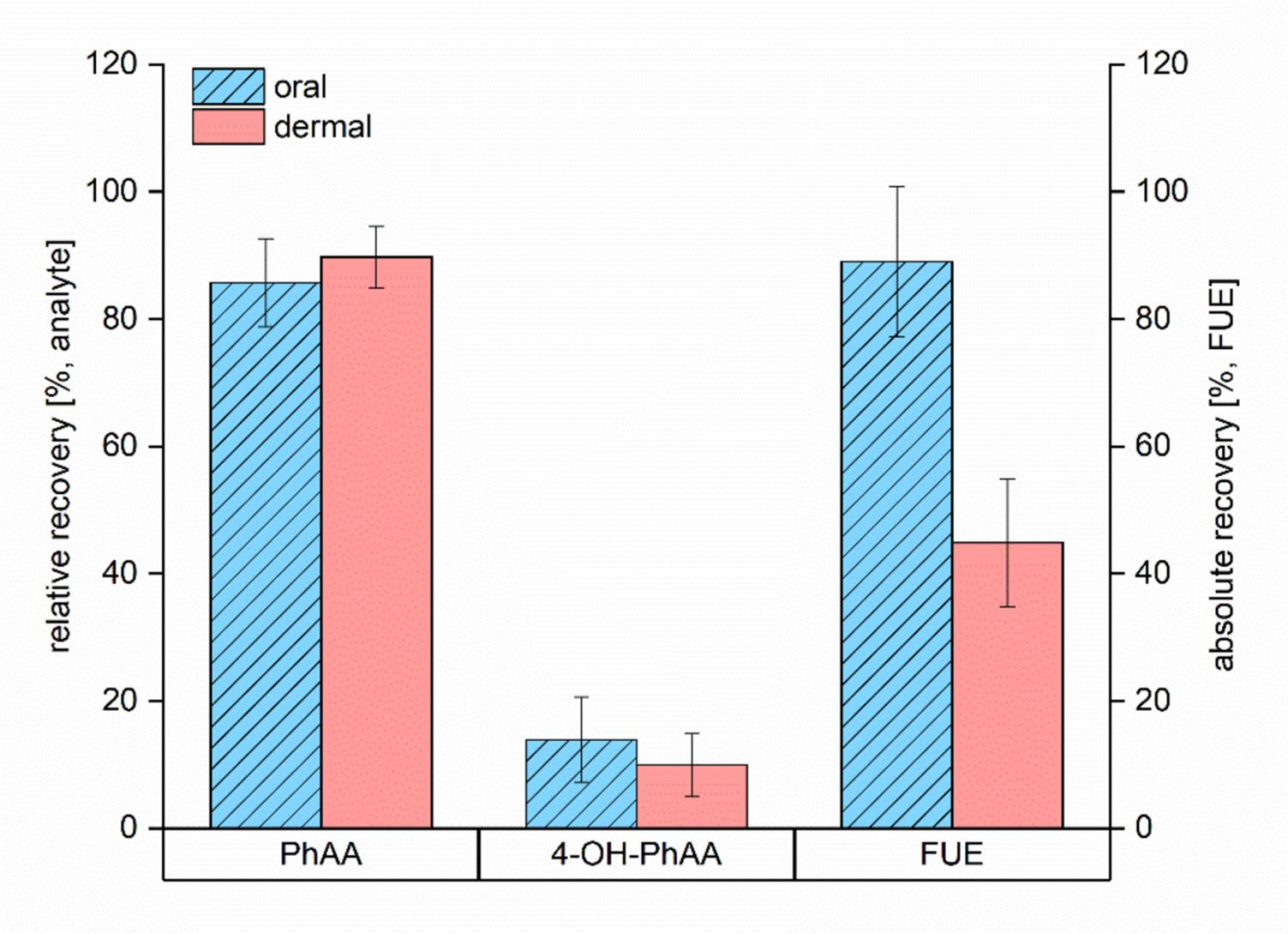
Relative shares of PhAA and 4-OH-PhAA as well as the absolute recovery rate (F_UE_) in urine following oral and dermal exposure to PhE to five volunteers, respectively (oral dose 5 mg/kg bw; dermal dose 0.4 mg/kg bw)

In the previous volunteer study of our working group, we investigated the metabolism and toxicokinetics of PhE following oral exposure, where five volunteers were orally exposed to about 5 mg PhE/kg bw each with the collection of urine and blood samples up to 48 h post-exposure (Eckert et al. 2024). There, we observed that resorbed PhE was almost exclusively excreted via urine within 48 h post-exposure (average conversion factor of 89.0 ± 11.8%). Accordingly, it may be assumed that the here observed dermal conversion factor of on average 45% presumably does correspond to the dermal resorption rate of PhE under the given exposure conditions. As illustrated in Fig. 3, the relative shares of the main metabolites PhAA and 4-OH-PhAA are very similar following oral or dermal exposure to PhE in five volunteers. In both studies, unmetabolized PhE and 4-OH-PhE were also confirmed to be excreted in urine following exposure to PhE, however, to a rather small extent of < 0.5% each, respectively (data not shown).

**Fig. 4:**
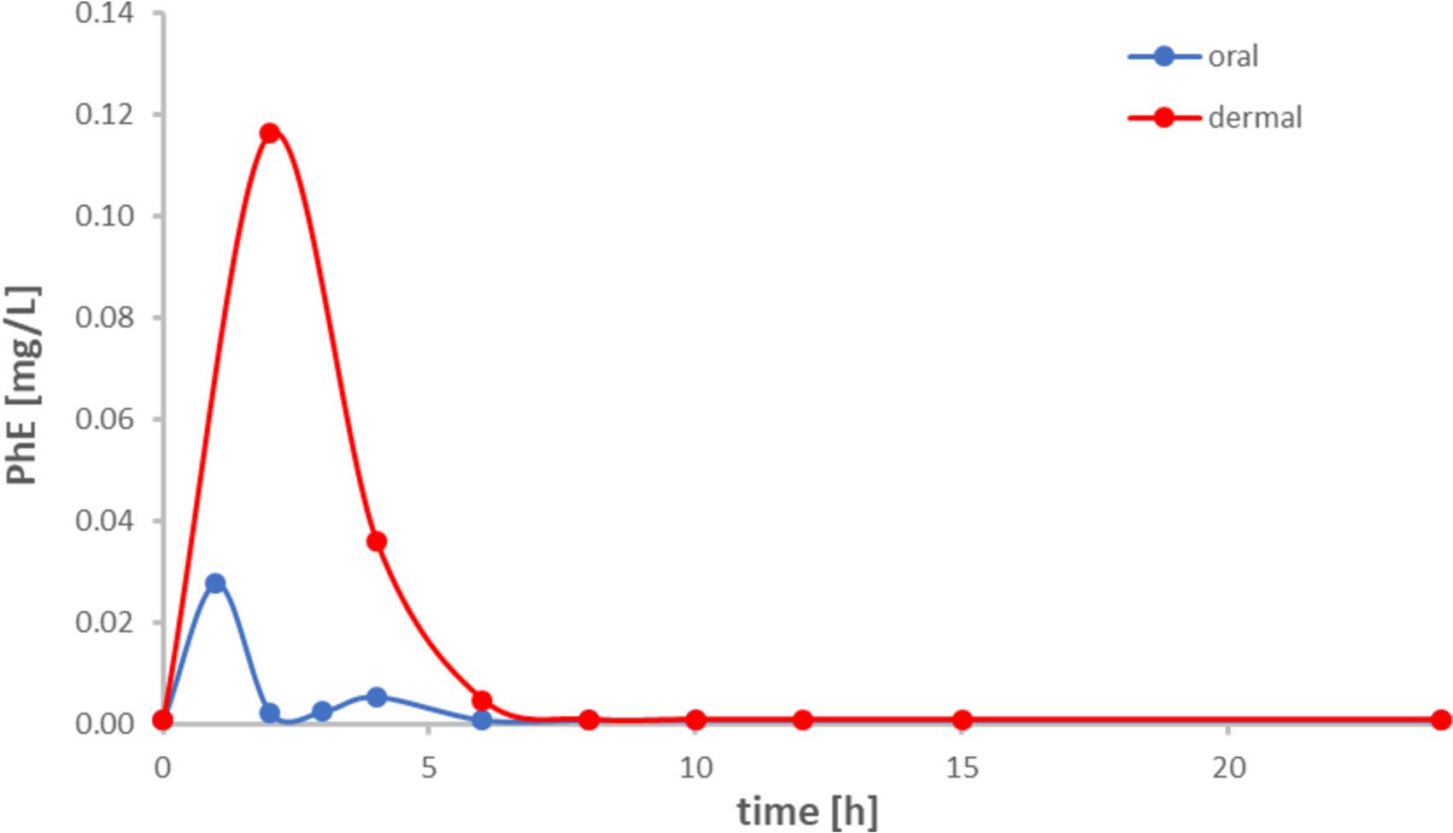
PhE levels in blood following oral and dermal exposure to about 5 mg PhE/kg bw each, respectively, to a single volunteer (based on data from Eckert et al. 2024)

The observed metabolism pattern of PhE following dermal and oral administration thus appears to be rather similar and may indicate low influence of both the dose and the route of PhE administration. Additionally, the observed half-lives in blood as well as in urine differ only marginally between the oral and the dermal study. Nevertheless, with regard to the applied administration route, we still identified some differences relating to the toxicokinetics of PhE and its metabolites:

- (1) The absolute urinary recovery rate (F_UE_) of PhE is significantly lower (by a factor of 2) following dermal exposure as compared to oral uptake (cf. Figure 3) indicating a lower resorption rate of PhE via human skin compared to gastrointestinal resorption. Previous studies, too, observed a lower resorption rate of PhE following dermal vs. oral exposure, especially when non-occlusive conditions were applied for dermal PhE exposure. However, the urinary recovery rates reported in several dermal studies—as well *in vitro* (using different animal skins) as *in vivo*—varied significantly from 37 % to about 85 % (Kim et al. 2015; Kwon et al. 2021; Roper et al. 1997; SCSS 2016; Stahl et al. 2011). In the only human study so far by Howes (1988) (poorly standardized), four hospitalized volunteers with different skin conditions were treated with a PhE-containing skin lotion. The total PhE recovery in urine was observed to vary considerably with levels between 8.5 and 42.1 % up to 72 h post-exposure. In the here presented study, the observed variation in the urinary PhE recovery following dermal PhE exposure of five volunteers was found to be much lower (44.9 ± 10.0 %) which might indicate a higher standardization regarding our chosen study design. Furthermore, the metabolic pattern and the half-lives of PhE and its metabolites were also observed to be similar following oral and dermal exposure. Thus, it may be reasonably assumed that the lower recovery in the dermal study is mainly due to a reduced resorption rate when realistic (i.e., non-occlusive) conditions are applied. The here observed urinary recovery rate of about 45 % of the dermal PhE dose thus now specifies the dermal bioavailability of PhE for humans.
- (2) As expected, systemic uptake of PhE via dermal penetration is delayed as compared to oral exposure that is presently illustrated by a significant postponement of the observed t_max_ levels following dermal exposure. This effect was already described for numerous other substances in humans (Oerlemans et al. 2019; Pluym et al. 2023; Stoeckelhuber et al. 2018, 2020). However, the observed effect may be considered as rather moderate for PhE compared to the majority of other compounds illustrating an extraordinarily rapid transdermal resorption of PhE, which may be explained by the amphiphilic character of PhE.
- (3) After dermal exposure, significantly higher levels of unmetabolized PhE in blood were observed in direct comparison to oral exposure (cf. Figure 4) indicating a considerable first-pass effect following oral exposure. This was already postulated by SCSS (2016) based on the results of animal studies with dermal and oral PhE exposure and was described by Eckert et al. 2024 based on a single comparison of oral and dermal PhE exposure in one volunteer. Accordingly, despite the reduction of the exposure dose of PhE used in the dermal study (0.4 mg/kg bw compared to 5.0 mg/kg bw in the oral study), the observed PhE levels in blood did not decrease equivalently (Figure 4). However, the observed effect can still be considered as rather minimal as the main metabolite in blood remains PhAA by a large margin. Using *in vitro* data, Hewitt et al. (2022) predicted tenfold higher c_max_ blood levels of PhAA compared to PhE for humans following PhE exposure. In our studies, however, we observed instead 477-fold and 34-fold higher PhAA levels in blood following oral (Eckert et al. 2024) and dermal exposure (this study), respectively.

As PhE is predominantly used in consumer products and in occupational settings, dermal exposure is presumed to be an important exposure route as well for the general population as under workplace conditions. Following dermal contact, PhE is rapidly taken up into the human body and mainly excreted in urine in form of the metabolites PhAA and 4-OH-PhAA. To monitor exposure to PhE, analysis of these two metabolites in urine may thus be recommended, accounting in sum for more than 99% of the renally excreted PhE dose. This is further supported by the study by Jäger et al. (2024) who analyzed 50 urine samples of the general population and observed considerable background levels of PhAA and 4-OH-PhAA in almost all samples, whereas 4-OH-PhE and unmetabolized PhE were only found in 36% and 32% of the analyzed samples, respectively. Interestingly, women showed higher mean and 95th percentile levels of all analytes in comparison to men, possibly due to the more frequent use of cosmetic products that often contain PhE as a preservative agent. Based on the here reported half-lives of PhAA and 4-OH-PhAA of about 4 h each, spot-urine samples should be collected directly after the end of exposure or at the end of a working-shift. Taking into account the dermal conversion factor (F_UE_) of PhE in the amount of 45% on average up to 48 h post-exposure (and 41% up to 24 h post-exposure), a recalculation of the PhE exposure dose is possible using 24-h urine samples. The oral exposure study, previously conducted by our working group did already show that virtually all resorbed PhE is excreted renally within 48 h. Thus, a monitoring of PhAA and 4-OH-PhAA in urine enables a direct estimation of the internal PhE exposure of the individuals investigated. Considering the short urinary elimination half-lives of PhE and its metabolites, the accumulation potential of PhE can be regarded as negligible.

## Conclusion

The present study demonstrates that PhE is rapidly resorbed at a relatively high rate by the human body following dermal exposure. It was shown to be quickly and extensively metabolized to the main metabolites PhAA and 4-OH-PhAA. Urinary elimination was found to be extensive and quite fast. Irrespective of the administration route, absorbed PhE is mainly excreted in urine in form of the acid metabolites PhAA and 4-OH-PhAA accounting together for over 99% of the renally excreted PhE dose. Taking into account the urinary elimination half-lives of 4.4 and 4.3 h for PhAA and 4-OH-PhAA, respectively, the good suitability of these two analytes as biomarkers of exposure to PhE is further supported. The urinary conversion factors following dermal PhE exposure of five volunteers amounted to 40.4 ± 9.8% and 4.4 ± 2.2% for PhAA and 4-OH-PhAA, respectively. As it was shown that virtually the entire absorbed PhE dose is excreted in urine within 48 h, a dermal resorption rate of PhE in humans (non-occlusive conditions) of about 45% can be derived.

The present study provides for the first time detailed insights into human biotransformation and toxicokinetics of PhE after dermal exposure and establishes a suitable strategy for human biomonitoring of PhE. The here presented results may thus be useful for further toxicokinetic modeling and forward dosimetry.

## Data Availability

The data that support the findings of this study are avialable from the corresponding author, Elisabeth Eckert, upon reasonable request.
